# *Satureja hortensis* L. and *Calendula officinalis* L., Two Romanian Plants, with In Vivo Antiparasitic Potential against Digestive Parasites of Swine

**DOI:** 10.3390/microorganisms11122980

**Published:** 2023-12-13

**Authors:** Mihai-Horia Băieş, Vlad-Dan Cotuţiu, Marina Spînu, Attila Mathe, Anamaria Cozma-Petruț, Vlad I. Bocǎneţ, Vasile Cozma

**Affiliations:** 1 Department of Parasitology and Parasitic Diseases, Faculty of Veterinary Medicine, University of Agricultural Sciences and Veterinary Medicine of Cluj-Napoca, 3-5 Mǎnǎştur Street, 400372 Cluj-Napoca, Romania; mihai-horia.baies@usamvcluj.ro (M.-H.B.); vlad.cotutiu@usamvcluj.ro (V.-D.C.); vasile.cozma@usamvcluj.ro (V.C.); 2 Department of Infectious Diseases, Faculty of Veterinary Medicine, University of Agricultural Sciences and Veterinary Medicine of Cluj-Napoca, 3-5 Mǎnǎştur Street, 400372 Cluj-Napoca, Romania; marina.spinu@gmail.com; 3 Agricultural Research and Development Station of Turda, Agriculturii Street, 27, 401100 Turda, Romania; mate_atta@yahoo.com; 4 Department of Bromatology, Hygiene, Nutrition, Faculty of Pharmacy, “Iuliu Haţieganu” University of Medicine and Pharmacy, 6 Pasteur Street, 400349 Cluj-Napoca, Romania; 5 Department of Manufacturing Engineering, Faculty of Industrial Engineering, Robotics and Production Management, Technical University of Cluj-Napoca, 400114 Cluj-Napoca, Romania; vlad.bocanet@tcm.utcluj.ro; 6 Academy of Agricultural and Forestry Sciences Gheorghe Ionescu-Siseşti (A.S.A.S.), Mărăști Boulevard, 61, 011464 Bucharest, Romania

**Keywords:** digestive parasites, pigs, *Calendula officinalis* L., *Satureja hortensis* L., low-input farms

## Abstract

Internal parasitic diseases of swine constitute a major welfare and health concern in low-input livestock farming. Due to an increase in chemical resistance, phytotherapeutic remedies have become an alternative for the prophylaxis and therapy of digestive parasitosis, albeit few remedies have been subjected to scientific validation. Low-input swine farming in Romania has adopted the traditional use of phytotherapy for controlling pathogens in livestock. The current study aimed to assess the antiparasitic potential of *Calendula officinalis* and *Satureja hortensis* against digestive parasites of swine in two low-input farms. The fecal samples were collected from sows, fatteners, and weaners, and were tested using the following coproparasitological methods: centrifugal sedimentation, flotation (Willis, McMaster egg counting technique), Ziehl–Neelsen stain modified by Henricksen, modified Blagg method, and in vitro nematode larvae/protozoan oocyst cultures. Six species of digestive parasites were diagnosed, namely *Ascaris suum*, *Trichuris suis*, *Oesophagostomum* spp., *Balantioides coli*, *Eimeria* spp., and *Cryptosporidium* spp., in various combinations, dependent on the swine category. A dose of 140 mg/kg bw/day of *C. officinalis* and 100 mg/kg bw/day of *S. hortensis* powders administered for 10 consecutive days revealed a strong antiprotozoal and anthelmintic activity on the aforementioned parasites. The curative efficacy can be attributed to the presence of polyphenols, sterols, tocopherols, and methoxylated flavones. In conclusion, our results indicate that *S. hortensis* and *C. officinalis* are promising alternatives to the commercially available antiparasitics, enabling their use as natural antiparasitic products against gastrointestinal parasites in pigs.

## 1. Introduction

The control of parasite infections in livestock farming is becoming increasingly important worldwide. Due to relatively high costs, most anthelmintic drugs are unavailable to rural subsistence livestock keepers. Furthermore, the large-scale use of anthelmintic drugs has led to various chemical resistance mutations [1,2]. Currently, benzimidazoles, imidazothiazoles, and macrocyclic lactones are commonly used for treating parasitic infections. A varying degree of resistance against these anthelmintics has been widely reported worldwide [3,4]. Residues of some such chemicals in the environment has the potential to disrupt the ecosystem, therefore, posing a significant threat to human health [4]. Consequently, a need to reduce the use of antibiotics and antiparasitics in livestock has shifted the spotlight onto phytotherapy [5,6]. Medicinal plants could present as an alternative to chemical molecules [4]. Phytotherapeutics are extracted from medicinal plants, while their bioactive compounds could be used to treat infectious and parasitic diseases [6,7]. The use of medicinal plants has seen a recent surge because of their lower toxicity and better biodegradability [1,6,8].

Pigs raised in conventional free-range systems appear to experience a higher standard of welfare (expressing a natural behavior, with access to outdoor areas, pasture, and enrichments) compared to pigs raised in conventional, indoor conditions [9]. On the other hand, the health risks associated with these systems (tail lesions, arthritis, skin lesions, bone fractures) can also be of concern [10]. Free-range access also contributes to the appearance of infectious and parasitic diseases [11]. Digestive parasites in swine affect reproductive output and swine performance (feed conversion, growth rate, and weight gain), with parasitized pigs being more prone to infectious and non-infectious diseases. This, in turn, undermines their gains in health and welfare [12]. Several reports attested the presence of gastrointestinal parasites in low-input farms, including protozoa (*Balantioides coli*, *Isospora suis*/*Eimeria* spp., *Cryptosporidium* spp., *Giardia* spp.) and nematodes (*Ascaris suum*, *Trichuris suis, Oesophagostomum spp*, and *Strongyloides ransomi*) [13,14,15,16]. Due to some anatomical and physiological similarities between pigs and humans, the former can serve as reservoirs for zoonotic pathogens, thus raising public health concerns [14,17]. Among such pathogens, *Trichinella spiralis*, *A. suum, Taenia solium, B. coli*, *C. parvum, Toxoplasma gondii*, *Sarcocystis suihominis*, *Entamoeba polecki, and Giardia duodenalis* are of particular interest [15,17,18,19,20,21].

Marigold, *Calendula officinalis* L., is an aromatic herb that belongs to the family Asteraceae and is used worldwide for its medicinal properties [22,23]. It also possesses several pharmacological activities such as the following: accelerating healing, neuroprotective, anti-inflammatory, hepatoprotective, antioxidant, immunostimulant, nephroprotective, hypoglycemic, gastroprotective, antibacterial, antifungal, antiviral, and insecticidal [22,23,24,25,26]. Due to the high concentration of saponins, *C. officinalis* showed antiparasitic activity [27,28]. The therapeutic properties of marigold are attributed to the presence of various classes of compounds, including, volatile oils (sesquiterpenoids monoterpenes), phenolic compounds (flavonoids and phenolic acids), coumarins, quinones, saponins, carotenoids, triterpenic alcohols, polyunsaturated fatty acids (calendic acid), polycarbohydrates, and other substances (proteins, amino acids, lipids saturated hydrocarbons, vitamin C, and mineral substances) [23,25,26,28,29]. *C. officinalis* triterpenoids are used as phytogenic feed additives which are sensory and flavoring compounds increasing growth performance, nutrient digestibility, and gut health in poultry [30,31].

*Satureja hortensis* L., known as summer savory, is an aromatic and medicinal plant belonging to the Lamiaceae family [32]. The major biomolecules found in extracts and essential oils of *S. hortensis* are volatile oils, phenolic compounds, flavonoids, tannins, steroids, acids, gums, mucilage, and pyrocatechols. The main components isolated from essential oils are carvacrol, thymol, cymene, terpinene, while alcoholic extracts are dominated by rosmarinic acid, caffeic acid, naringenin, isoferulic acid, luteolin, quercetin, and apigenin [33,34,35,36]. These bioactive compounds have been found to have a variety of biological activities including antioxidant, antispasmodic, anti-inflammatory, analgesic, antidiabetic, hepatoprotective, immunostimulant, reproduction stimulatory, vasodilatory, antimicrobial, and antiparasitic [32,34,37,38]. Carvacrol and thymol are believed to be responsible for the antiprotozoal and anthelmintic effects of *S. hortensis*.

These two plants were included in empirical therapies because of their antiparasitic effect. The current study aimed to evaluate the in vivo antiparasitic activity of *C. officinalis* and *S. hortensis* powders against digestive parasites in swine, in two low-input farms from the Transylvania area.

## 2. Materials and Methods

### 2.1. Chemical Analysis of Satureja hortensis and Calendula officinalis

The aerial parts of both *S. hortensis* and *C. officinalis* were utilized. Analysis of the bioactive compounds present in studied plants was conducted using high performance liquid chromatography coupled with mass spectrometry (HPLC-MS). All experimental procedures were performed at the Iuliu Haţieganu University of Medicine and Pharmacy, in Cluj-Napoca. Detailed information about the machines, methods, and techniques employed for analyzing the ethanolic extracts of marigold and summer savory can be found in a previous study [39].

### 2.2. Experimental Design

Before initiating the experiment, a preliminary study was carried out on a limited number of animals. During this pilot study, various doses (in accordance with existing literature), of *S. hortensis* and *C. officinalis* were administered, and their effects evaluated. The animals’ feeding behavior, the plants’ antiparasitic effectiveness, and any possible adverse reactions were meticulously observed.

The same two low-input farms (F1 and F2), as previously described in [16], were used to provide samples. F1 had a pig herd of 420 animals while F2 had 305 animals. The study was initiated in April 2022, and concluded in July 2022. The farms included in the experiment were homogeneous in terms of rearing system, geographic location, pig breeds raised, feed used, and identified parasites.

*S. hortensis* and *C. officinalis* were obtained from Romanian flora (local sources) by an authorized company who provided the plants. The aerial parts of both plants were ground, resulting in a feed containing either marigold or summer savory. Each type was then mixed with cereal flour. The study was conducted on both farms, with Bazna and Mangalitza breed pigs equally distributed in each group. A total of 240 pigs were included in the study, with 120 pigs assigned to each plant-based experiment variant on both farms. Three control groups (10 weaners, 10 fatteners, and 10 sows) and another three experimental groups (10 weaners, 10 fatteners and 10 sows) were established for each farm and plant in the experiment. Consequently, 60 pigs were used for the marigold experiment, with the same number used for the summer savory experiment, amounting to a total of 120 individuals per farm. The sows (S) were aged from 1 to 4 years with a body weight of 135 to 140 kg, fatteners (F) were aged 5 to 6 months and weighing 55 to 60 kg, and weaners (W) were aged 11 to 12 weeks and weighing 12 to 14 kg. Ten individuals, of the same age and weight, were confined in a pen, constituting an experimental group (EG). Welfare standards were met while administering the feed, diets being tailored to the animals based on their respective age categories (Table 1). Daily feed intake averages per pig were 0.8 kg for weaners, 2.5 kg for fatteners, and 3.5 kg for sows. The EG received a dose of 100 mg/kg bw/day of *S. hortensis* powder divided into two portions for a total period of ten consecutive days, while *C. officinalis* powder was administered in a dose of 140 mg/kg bw/day, identically to summer savory. The study started by testing *S. hortensis* for a period of 28 days, followed by *C. officinalis* for the same amount of time. A one-and-a–half-month period between the experiments conducted on different individuals was also included in the protocol. For each farm, swine category, and plant, three coproparasitological examinations (day 0 = before therapy, day 14 and day 28 = after therapy) were performed.

The fecal samples were collected individually (weighing approximately 15–20 g each), placed in sterile containers, examined for the presence of macroscopic parasites, then numbered and stored at a temperature of 2–8 °C for up to 48 h, until further examination. The collected samples were tested using the following coproparasitological methods: centrifugal sedimentation, flotation (Willis, McMaster egg counting technique), Ziehl–Neelsen stain modified by Henricksen, modified Blagg method, and in vitro nematode larvae/protozoan oocyst cultures [16,40,41].

### 2.3. Ontologies, Ethics Statement, and Assessment of Antiparasitic Efficacy

Table S1 provides a comprehensive description of the ontologies related to medicinal plants, chemical compounds, parasites, and diseases, used in the present study.

The behaviour, welfare, and clinical condition of the pigs were continuously monitored before and during the experiment. The conducted study adhered to both national regulations (Law No. 43 of 2014) and European (EU Directive No. 63 of 2010) legislation concerning bioethical rules of experimentation on animals.

To evaluate the antiparasitic efficacy of *C. officinalis* and *S. hortensis,* a fecal egg count reduction test (FECRT) was performed. The methodology employed for this test was described by McKenna (2006) [42] and was also utilized in a previous study [43].

### 2.4. Statistical Analysis

The descriptive analysis was performed using Python 3.9.17 with the SciPy 1.11.1 package [44] and the visualizations using the Seaborn 0.12.2 library [45]. The inferential analysis was conducted using Python/SciPy and IBM SPSS v26 [46].

The first step was to analyze the data from a descriptive point of view. First, the evolution of parasites over time was evaluated for each parasite type and treatment regardless of pig type. Missing values were excluded from the analysis.

Considering that values were collected for two treatment groups for three time points, a repeated measures two-way ANOVA was considered to be the appropriate test. This test makes the following assumptions about the data:

- Sphericity: The variances of the differences between all combinations of related groups (levels) are equal.
- Normality: The distribution of the differences in the dependent variable between the two related groups should be approximately normally distributed for each level of the independent variable.
- Lack of multivariate outliers.

Sphericity was tested using the Mauchly test of sphericity and the normality with the Shapiro–Wilk test. Both assumptions were violated even after data was transformed (using logarithmic or square root transforms) or outliers removed. As a result, non-parametric tests were used as they do not make assumptions about the shape of the distribution. Because the non-parametric alternative to repeated measures ANOVA (the Friedman test) does not accommodate a between-subjects factor directly, the following alternative approach was used:

1. For the within-subjects factor (time) the Friedman test was used to compare the repeated measures (measurements at day 0, 14 and 28) for each group separately (control and experimental group). If the Friedman test returned significant differences, the Wilcoxon signed-rank test for pairwise comparisons was used to identify the groups with the significant differences. The Bonferroni correction for multiple comparisons was used to reduce the risk of false positives.
2. For the between-subjects factor (treatment group) a Mann–Whitney U test was performed for each time point to compare the control and experimental groups.

When looking at the sample counts, values varied from 10 results for *T. suis* (TS) with CO for both farms (F1, F2) and all types of pigs (S, F, W) to 60 results for *Eimeria* spp. (ES), and *B. coli* (BC). Only pairs that contain more than 30 values for both farms were considered for the analysis to increase the chances of reproductible results. The analysis was conducted on results from pigs regardless of farm and type. The analyzed parasites were as follows: ES, BC, *A. suum* (AS), and *Cryptosporidium* spp. (CR).

## 3. Results

### 3.1. Chemical Analysis of S. hortensis and C. officinalis

Four major compounds were identified following the chemical analysis of the *S. hortensis* and *C. officinalis* ethanolic extracts: polyphenols (chlorogenic acid, caffeic acid, p-coumaric acid, ferulic acid, isoquercitrin, rutoside, quercitrin, quercetol, luteolin, apigenin, syringic acid, protocatechuic acid, vanilic acid), tocopherols (α-tocopherol, γ-tocopherol, Δ-tocopherol), sterols (ergosterol, stigmasterol, Β-sitosterol, campesterol) and methoxylated flavones (jaceosidin, hispidulin, acacetin) for summer savory and polyphenols (chlorogenic acid, isoquercitrin, rutoside, quercitrin, syringic acid, protocatechuic acid, vanilic acid), (α-tocopherol, γ-tocopherol, Δ-tocopherol), and sterols (ergosterol, stigmasterol, Β-sitosterol, campesterol) for marigold.

### 3.2. Analysis of Antiparasitic Effects of Studied Plants

The animals consumed the feed without hesitation and without any side effects or toxicity observed. In fatteners and sows treated with summer savory, a vermifuge effect was noticed, indicated by the elimination of *A. suum* adults through feces. Throughout the entire experiment, the welfare and health of the pigs were well maintained.

The coproparasitological examination showed co-infections with protozoa and nematodes. Six species of digestive parasites were diagnosed: *Ascaris suum, Trichuris suis, Oesophagostomum* spp. (OE), *Balantioides coli, Eimeria* spp., and *Cryptosporidium* spp., in variable combinations depending on the category of swine. The prevalence and average intensity of parasitic infections varied depending on each farm, age category, and studied plant. In sows, *A. suum*, *Oesophagostomum* spp., *B. coli,* and *Eimeria* spp. were encountered in both farms, while in F2, *Cryptosporidium* spp. was additionally found. In fatteners, *A. suum*, *T. suis*, *B. coli,* and *Eimeria* spp. were identified in both farms. In weaners from both farms, *Cryptosporidium* spp., *B. coli,* and *Eimeria* spp. were diagnosed, while *Oesophagostomum* spp. was additionally identified in F2 (Tables S2–S4).

Both summer savory and marigold were effective against all diagnosed parasites, with the exception of *Cryptosporidium* spp. Neither plant showed any antiprotozoal activity on *Cryptosporidium*. However, *S. hortensis* demonstrated a pronounced anthelmintic and antiprotozoal effect comparative with *C. officinalis*. Overall, the maximum therapeutic effects of summer savory and marigold varied according on the farm, age category, and day of examination.

The therapeutic efficacy (reduction%) of *S. hortensis* (SH) and *C. officinalis* (CO) on identified parasites across all age groups is detailed in Table 2. For *Cryptosporidium* only the prevalence was calculated because the classic coproparasitological methods used were not able to quantify the number of oocysts.

#### 3.2.1. Descriptive Statistics

**Figure 1:**
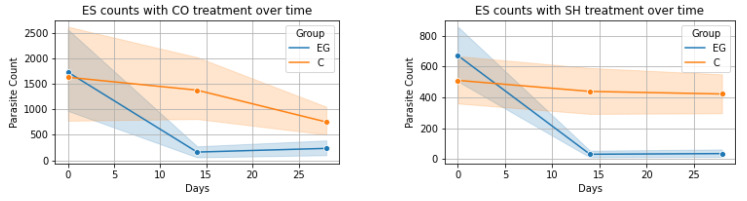

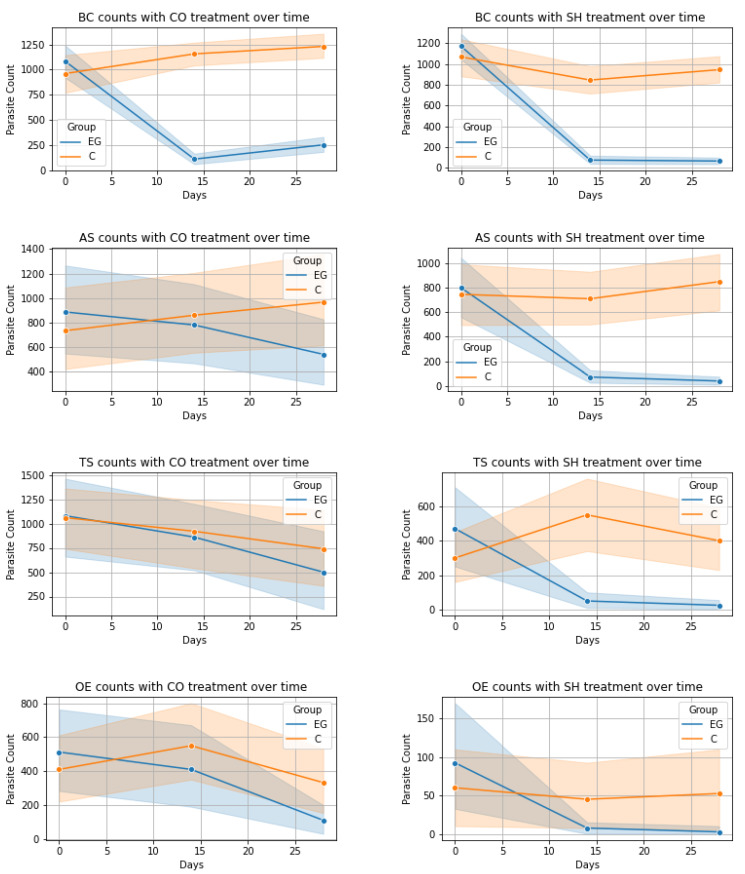
Evolution of parasite count over time for each parasite-treatment pair: EG—experimental group, C—control group; ES = *Eimeria* spp., BC = *B. coli*, AS = *A. suum*, TS = *T. suis*, OE = *Oesophagostomum* spp., CO = *Calendula officinalis*, SH = *Satureja hortensis*.

The evolution of the numbers of oocysts, cysts, and eggs per gram of fecal matter was visualized for each analyzed time point (Figure 1) containing the 95% confidence intervals. In the case of ES and BC, both treatments (CO and SH) had very distinct evolution on days 14 and 28. This is also true for the AS and TS in the treatment with SH but when treated with CO, the confidence intervals had a wide overlap indicating less distinct results. In the case of OE, both treatments seemed to have less of an effect.

#### 3.2.2. Inferential Statistics

In this study, we assessed the impact of different treatments on parasite levels using a range of non-parametric statistical methods.

**ES-CO Treatment**: The Friedman test applied to the control group for ES parasite levels under CO treatment showed no significant variations over time (χ^2^(2) = 2.65, *p* = 0.265). However, the experimental group exhibited a notable change (χ^2^(2) = 24.61, *p* < 0.001). Wilcoxon signed-rank test comparisons in the control group revealed a significant decrease only between Day 14 and Day 28 (W = 202.5, *p* = 0.040), with no significant changes in other comparisons. In contrast, the experimental group saw significant decreases from Day 0 to Day 14 and Day 0 to Day 28 (W = 47.5 and W = 53.5, both *p* < 0.001), but not between Day 14 and Day 28. The Mann–Whitney U test indicated significant differences between the groups on Day 14 and Day 28 (U = 734.5 and U = 796.5 respectively, both *p* < 0.05).

**ES-SH Treatment**: Similar analysis for the SH treatment showed no significant changes in the control group (χ^2^(2) = 0.68, *p* = 0.711), unlike the experimental group which demonstrated significant changes (χ^2^(2) = 55.50, *p* < 0.001). Wilcoxon tests in the experimental group revealed significant decreases in ES levels from Day 0 to Day 14 (W = 7.00, *p* < 0.001) and from Day 0 to Day 28 (W = 10.50, *p* < 0.001), with no change between Day 14 and Day 28 (W = 46.00, *p* = 0.680). The Mann–Whitney U test showed significant disparities between the groups on Day 14 and Day 28 **(U = 1058.5 and U = 982.5 respectively, both *p* < 0.001)**.

**BC-CO and BC-SH Treatments**: For both CO and SH treatments on BC parasite levels, Friedman tests in control groups indicated no significant changes. However, experimental groups showed pronounced shifts (CO: χ^2^(2) = 70.09; SH: χ^2^(2) = 94.54, both *p* < 0.001). Wilcoxon tests revealed various significant changes at different time points in experimental groups. In the experimental group, there were significant reductions in BC levels from Day 0 to Day 14 (W = 4.00, *p* < 0.001) and from Day 0 to Day 28 (W = 58.50, *p* < 0.001) with a slight but significant increase from Day 14 to Day 28 (W = 129.50, *p* = 0.004) in the CO treatment. Similarly, a significant decrease was observed in the SH experimental group between Day 0 and Day 14 and Day 0 and Day 28 (W = 2.00 and W = 1.00 respectively, both *p* < 0.001). Mann–Whitney U tests highlighted significant differences between control and experimental groups at later stages, Day 14 **(U = 233.00, *p* < 0.001 for CO and U = 3017.50, *p* < 0.05 for SH) and Day 28 (U = 221.5, *p* < 0.001 for CO and U = 3324.50, *p* < 0.05 for SH)**.

**AS-CO and AS-SH Treatments**: The impact of CO and SH treatments on AS parasite levels was also analyzed. Friedman tests showed no significant changes in control groups, in contrast to significant alterations in experimental groups (χ^2^(2) = 7.80, *p* = 0.020 for CO and χ^2^(2) = 29.35, *p* < 0.001 for SH). Wilcoxon tests within the experimental groups revealed significant reductions at various time points, more specifically from Day 14 to Day 28 (W = 11.00, *p* = 0.004) and Day 0 to Day 28 (W = 21.00, *p* = 0.008) for CO, and from Day 0 to Day 14 (W = 3.00, *p* < 0.001) and Day 0 to Day 28 (W = 11.50, *p* < 0.001) for SH. Mann–Whitney U tests indicated no significant differences between the groups at most time points with the exception of Day 14 (U = 375.00, *p* < 0.001) and Day 28 (U = 282.0, *p* < 0.001) for the SH treatment.

**CR-CO and CR-SH Treatments**: For dichotomous CR parasite data, Friedman tests in both CO and SH treatments revealed no significant changes over time in either control or experimental groups. Chi-squared and Fisher’s exact tests for both treatments showed no significant differences between control and experimental groups at all time points.

## 4. Discussion

Over the past decades the use of local medicinal plants and non-chemical molecules instead of chemical drugs to treat parasitic diseases in humans and animals has experienced a resurgence [47]. Phytotherapeutic remedies are considered sustainable and adaptable to rural farming communities due to their availability and simplicity of preparation and administration to animals [48]. The therapeutic use of medicinal plants is reportedly widespread in humans, while limited in animals [49]. The aim of the current study was to assess the antiparasitic potential of two Romanian plants (*S. hortensis* and *C. officinalis*) against digestive parasites of swine. The major compounds of summer savory and marigold (polyphenols, sterols, tocopherols, and flavones) possess both in vivo and in vitro anthelmintic and antiprotozoal effects [50,51,52,53].

No studies about the dose of *C. officinalis* in pigs have been reported. Therefore, we extrapolated results from other animal species, including broiler chickens (150–450 mg/kg), rats (50–6000 mg/kg), mice (250–5000 mg/kg), rabbits (6000 mg/kg), and guinea pigs (250–500 mg/kg) [54,55,56,57,58]. In the current study, we elected to use a dose of 140 mg/kg bw/day, divided into two portions, administered for ten consecutive days. *C. officinalis* was found to be non-toxic, non-mutagenic, and non-genotoxic lacking reports of mortality [23,59]. In rare cases, marigold can cause allergic reactions on the skin, low hepatic toxicity on chronic exposure in rats, and in a rare report, anaphylactic shock [28,60,61]. The use of summer savory in swine diet and the proper dosage have been understudied. Therefore, we focused our attention onto the existing studies on humans (250–500 mg/individual/day) and various other animal species. These included rabbits (250 mg/kg/day), rats (500–5000 mg/kg/day), and broiler chicks (100–400 mg/kg/day) [32,62,63,64,65,66]. In the present study, the elected dose of *S. hortensis* powders was 100 mg/kg bw/day, administered under the same protocol as for marigold. Summer savory is considered a safe plant both for humans and animals, with very few side-effects, but it should be used with caution by patients with diabetes, hypoglycemia, hypertension or bleeding disorders, and is not recommended for children and pregnant women due to a lack of sufficient evidence for its use [32,33].

*Ascaris suum* is one of the most prevalent gastrointestinal parasites in domestic pigs widespread worldwide and causes significant economic losses in the swine industry [67,68]. In the present study, *A. suum* was diagnosed in fatteners and sows, in both farms. *C. officinalis* was variably effective against this parasite (10.3–79.9%), dependent on age group, while *S. hortensis* possesses a relatively strong anthelmintic activity range between 59.7% and 91.1%.

*Oesophagostomum* spp. is a large intestinal geohelminth—infections tend to be subclinical inducing weight loss of sows, low birth rates, and reduced growth of piglets [69]. *Oesophagostomum* was identified in weaners and sows. Both plants were effective against it, but summer savory (69.2–100%) was superior to marigold (28.6–60.5%).

*Trichuris suis*, the swine whipworm, is a widespread geohelminth with common manifestations including diarrhea, anorexia, retarded growth, and performance losses [70]. *T. suis* was diagnosed only in fatteners, in both farms. *S. hortensis* was very effective (80.5–90.3%) against *T. suis*, while *C. officinalis* had a weak anthelmintic activity range between 8.2% and 20.3%.

*Cryptosporidium* is a zoonotic protozoa of pigs, which causes diarrhea, particularly in immunodeficient individuals and children [71]. *Cryptosporidium* spp. was identified in weaners and sows, in both farms. Neither marigold nor summer savory were effective against this parasitic infection.

*Balantioides coli* is a ciliat-comensal protozoan which can be transmitted from pigs to humans and act as an occasional pathogen [72]. *B. coli* was diagnosed in both farms, in all swine categories. Both plants were very effective against *B. coli*. Efficacy ranged between 53.6% and 90.9% for marigold and between 63.5% and 88.4% for summer savory. No other studies about the efficacy of these two plants against *B. coli* have been reported.

*Eimeria* species are common in pigs worldwide, occasionally clinically affecting weaners and fatteners when diarrhea and weight loss can be observed upon infection with the more pathogenic species [73]. *Eimeria* was identified in all age categories, in both farms. Both plants demonstrated a strong antiprotozoal activity, ranging between 30.0% and 95.5% for *C. officinalis* and between 25.1% and 94.1% for *S. hortensis*, respectively.

The antiparasitic activity of marigold is very poorly understood. There are several reports on the effectiveness of this plant on protozoa such as: *Plasmodium falciparum, Hexamita muris, Trichomonas spp., Chilomastix bettencourti, Leishmania* spp., and also on helminths including *Heligmosomoides polygyrus* and *Heligmosomoides bakeri* [51,74,75,76,77]. Acaricidal and insecticidal activity was demonstrated against *Sarcoptes scabiei*, *Rhipicephalus microplus,* and *Oncopeltus fasciatus* [78,79,80]. Triterpenoid saponins, oleanic acid, and its glycosides are bioactive compounds isolated from marigold which are responsible for the antiparasitic activity [50,51]. A diet rich in carotenoids seems to promote resistance against oocysts of *Eimeria* and the excretion of a massive number of oocysts is attenuated or delayed. Effects included a reduction in the severity of coccidiosis symptoms as well as a delay in the parasite’s life cycle, reducing the load of oocysts in feces [1,81].

*Satureja hortensis* alcoholic extracts and essential oils have demonstrated antiprotozoal and anthelmintic effects against *Ascaris* spp., *Trichuris muris*, *Cryptosporidium* spp., *Eimeria* spp. and digestive strongyles of ruminants. A detailed list about the antiparasitic activities of *S. hortensis* is presented in Table 3. Other studies have highlighted the antiparasitic properties of *Satureja* species against *Echinococcus granulosus, Leishmania* spp., *Plasmodium spp., Trypanosoma spp., Giardia lamblia, Trichomonas vaginalis, Toxoplasma gondii,* and *Acanthamoeba castellanii* [82,83,84,85,86,87,88,89,90].

Improving the feeding value of swine diets through supplementation of bioactive and high nutritional medicinal plants such as summer savory and marigold is a viable approach to enhance the supplement utilization effectiveness for increasing productivity. Furthermore, it increases the quality of the animal products obtained via sustainable livestock production systems. Data regarding effects of different medicinal plants against gastrointestinal parasites of swine are scarce. The mechanism behind the antiprotozoal and anthelmintic effects of studied plants is still unknown. The current study and other reports revealed appropriate antiparasitic activity of *S. hortensis* and *C. officinalis* which indicate that both plants offer a viable and sustainable alternative to classic antiparasitics, enabling their use as an alternative to, or in addition to chemical drugs in parasite control programs.

## 5. Conclusions

The current study demonstrated the efficacy of powdered *C. officinalis* and *S. hortensis* aerial parts against digestive parasites in pigs when administered at doses of 140 mg/kg/day and 100 mg/kg/day, respectively, over a period of 10 consecutive days.

Considering the data presented in this study, summer savory and marigold powders showed promising in vivo antiparasitic activity and, therefore, might be used as antiparasitic natural products after scientific validation. *C. officinalis* had a strong antiprotozoal activity and mildly anthelmintic effects while *S. hortensis* was very effective against both helminths and protozoa infections.

Nevertheless, our discovery attests that summer savory and marigold are accessible antiparasitic remedies and can be used as an alternative therapy to chemical drugs against parasitic infections in swine, prompting the development of phytomedicine. Moreover, the current study is the first report about the antiparasitic effects of *C. officinalis* and *S. hortensis* against digestive parasites of swine, from Romania.

However, further studies are required to determine the bioactive compounds responsible for the antiparasitic activity, the potential toxic reactions, the minimum effective dosage, and the frequency of the administration for each plant.

## Figures and Tables

**Table 1:** The diet for the EG tailored according to specific age groups.

Feed	Summer Savory Group			Marigold Group		
	Sows	Fatteners	Weaners	Sows	Fatteners	Weaners
Calcium carbonate %	1.4	1.4	1.4	1.4	1.4	1.4
Peas %	15	15	10	15	15	10
Wheat %	25	25	20	25	25	20
Barley %	20	12	30	20	12	30
Corn %	38.20	46.36	38.43	38.04	46.27	38.36
Aerial parts of *S. hortensis* %	0.40	0.24	0.17	-	-	-
Aerial parts of *C. officinalis* %	-	-	-	0.56	0.33	0.24

**Table 2:** The percentage of reduction in fecal egg/oocyst/cyst count (%) registered on days 14, and 28 post-treatment in F1 and F2 farms (applying FECR formula).

Parasite	*C. officinalis* (14)						*C. officinalis* (28)					
	Weaners		Fatteners		Sows		Weaners		Fatteners		Sows	
	F1	F2	F1	F2	F1	F2	F1	F2	F1	F2	F1	F2
*A. suum*	-	-	15.2	10.3	-	49.9	-	-	54.2	34.9	-	79.9
*T. suis*	-	-	-	8.2	-	-	-	-	-	20.3	-	-
*Oesophagostomum* spp.	-	60.5	-	-	-	28.6	-	32.9	-	-	-	45.8
*Eimeria* spp.	91.8	42.5	95.5	75.9	-	74.9	72.5	57.1	88.9	30.0	-	76.5
*B. coli*	72.0	90.9	73.1	53.6	84.9	69.8	74.7	69.2	58.3	61.1	76.1	58.2
**Parasite**	***S. hortensis* (14)**						***S. hortensis* (28)**					
	**Weaners**		**Fatteners**		**Sows**		**Weaners**		**Fatteners**		**Sows**	
	**F1**	**F2**	**F1**	**F2**	**F1**	**F2**	**F1**	**F2**	**F1**	**F2**	**F1**	**F2**
*A. suum*	-	-	70.8	77.1	91.1	88.7	-	-	77.1	81.2	72.1	59.7
*T. suis*	-	-	80.5	84.0	-	-	-	-	90.3	87.1	-	-
*Oesophagostomum* spp.	-	-	-	-	80.2	69.2	-	-	-	-	100	83.7
*Eimeria* spp.	78.2	68.7	76.3	89.7	25.1	70.3	66.8	80.3	46.8	83.8	80.9	94.1
*B. coli*	80.1	88.4	63.5	74.7	70.2	70.5	83.6	86.5	72.2	71.2	70.7	74.6

“-“ = was not diagnosed.

**Table 3:** The antiparasitic activity of *Satureja* species (literature reports).

Parasite	Bioactive Compound/ Extract	Evaluation	Antiparasitic Activity	References
*A. suum*	ethanolic extract	in vitro	ovicidal and development eggs inhibition	[91]
*A. suum*	a-pinene/p-cymene/ thymol octanoate	in vivo	reduction of total worm counts, female worm counts, fecal egg counts, and worm volume	[52]
*A. suum*	carvacrol	in vitro	inhibitory effect on the contractions induced by acetylcholine	[92]
*Parascaris univalens*	carvacrol	in vitro	paralytic and lethal effects on L3 stage larvae	[93]
*Parascaris* spp.	carvacrol	in vitro	fully and irreversibly abolished adult parasite muscle contractions	[94]
*Trichuris muris*	thymol	in vitro	inhibition of the motility of the adult worms	[95]
*Eimeria* spp.	thymol/carvacrol/ saponins	in vitro	decreasing the effectiveness of *Eimeria* sporozoites to invade bovine cells	[96]
*Eimeria* spp.	carvacrol/thymol	in vitro	destruction of oocysts	[97]
*Eimeria* spp	thymol	in vivo	reducing oocysts shedding and intestinal lesions	[98]
*Cryptosporidium parvum*	carvacrol/thymol/thymol octanoate	in vitro	inhibition of parasitic invasion and infection of human cells	[99,100]
*Cryptosporidium*	thymol	in vivo	reduces the pathogen parasitic load	[101]
*Cryptosporidium baileyi/Cryptosporidium galli*	thymol	in vitro	destructive effect on oocysts at very low concentrations	[102]
*Haemonchus contortus/Trichostrongylus/Teladorsagia*/*Chabertia*	thymol/carvacrol/p-cymene/terpinene	in vitro/in vivo	inhibition of egg hatching/larval development and larval motility/reduction of fecal eggs count	[7,103,104]
*Trichostrongylus colubriformis*	ethanolic extract	in vitro	migration inhibition of the infective third-stage larvae	[91]

## Data Availability

Data are contained within the article and Supplementary Material.

## Supplementary Materials

The following supporting information can be downloaded at: https://www.mdpi.com/article/10.3390/microorganisms11122980/s1, Table S1: Ontologies/medicinal plants, chemical compounds, pathogens, and diseases, used in experiment; Table S2: The prevalence (P), at 0, 14, and 28 days in weaners; Table S3: The prevalence (P), at 0, 14, and 28 days in fatteners; Table S4: The prevalence (P), at 0, 14, and 28 days in sows.
