# Supplementary material for: Satureja hortensis L. and Calendula officinalis L., Two Romanian Plants, with In Vivo Antiparasitic Potential against Digestive Parasites of Swine
Source: Microorganisms. 2023 Dec 13;11(12):2980. doi: 10.3390/microorganisms11122980 (PMC10746069; doi:10.3390/microorganisms11122980)
Supplement: Supplementary file 1 [file microorganisms-11-02980-s001.zip › microorganisms-2741452-supplementary.pdf]

Supplementary Data

Table S1. Ontologies/ medicinal plants, chemical compounds, pathogens, and diseases, used in experiment.

**Traits**	ATOL*, AHOL**, OPL***, IPNI****, and ChEBI***** References	
Medicinal plants used	*Calendula officinalis* L	187894-1
	Satureja hortensis L.	457680-1
Chemical compounds detected	Polyphenols (μg/mL)	26195
	Tocopherols (ng/mL	135821
	Methyoxylated flavones (ng/mL)	25241
	Sterols (μg/mL)	15889
Parasite load traits	Parasite Oocysts (OPG)	Oocyst Stage
	Parasite Cysts	Cyst Stage
	Parasite Eggs (EPG)	Egg Stage
Parasite used	*Eimeria* spp.	AHOL_0004070
	Balantioides coli	AHOL_0004016
	Ascaris suum	AHOL_0004179
	Trichuris suis	AHOL_0004186
	Oesophagostomum spp.	AHOL_0004181
	Cryptosporidium spp.	AHOL_0004175
Disease description	Ascaridiosis	AHOL_0005382
	Coccidiosis	AHOL_0005374
	Cryptosporidiosis	AHOL_0005377

*Traits in reference to the ontology ATOL: https://www.atol-o.com/en/atol-2/; **Traits in reference to the ontology AHOL: https://www.atol-onntologytology.com/ahol/; *** Ontology for Parasite Life cycle: http://wiki.aiisc.ai/index.php/Ontology_for_Parasite_Life_Cycle; **** International Plant Names Index-IPNI : *https://www.ipni.org/p/3;* ***** Chemical Entities of Biological Interest-ChEBI: https://www.ebi.ac.uk/chebi/.

Table S2. The prevalence (P) at 0, 14 and 28 days in weaners.

			**F1**			
	**C (0)**	**CO (0)**	**C (14)**	**CO (14)**	**C (28)**	**CO (28)**
**Parasite**	P %	P %	P %	P %	P %	P %
Eimeria spp.	20	30	40	10	50	20
B. coli	80	80	100	30	100	50
Cryptosporidium spp.	20	20	20	10	30	20
	**C (0)**	**SH (0)**	**C (14)**	**SH (14)**	**C (28)**	**SH (28)**
**Parasite**	P %	P %	P %	P %	P %	P %
Eimeria spp.	60	70	50	20	40	20
B. coli	90	100	80	10	90	30
Cryptosporidium spp.	20	30	20	30	10	10
			**F2**			
	**C (0)**	**CO (0)**	**C (14)**	**CO (14)**	**C (28)**	**CO (28)**
**Parasite**	P %	P %	P %	P %	P %	P %
Eimeria spp.	100	90	80	40	80	40
B. coli	80	90	90	30	100	90
Oesophagostomum spp.	40	50	50	20	40	30
Cryptosporidium spp.	20	30	20	20	10	10
	**C (0)**	**SH (0)**	**C (14)**	**SH (14)**	**C (28)**	**SH (28)**
**Parasite**	P %	P %	P %	P %	P %	P %
Eimeria spp.	80	90	70	20	80	20
B. coli	90	90	70	50	100	30
Cryptosporidium spp.	20	20	20	10	10	10

F=farm, C=control, SH=*S. hortensis*, CO=*C. officinalis*

Table S3. The prevalence (P), at 0, 14 and 28 days in fatteners.

F1						
	**C (0)**	**CO (0)**	**C (14)**	**CO (14)**	**C (28)**	**CO (28)**
**Parasite**	P %	P %	P %	P %	P %	P %
Eimeria spp.	20	30	40	20	50	30
B. coli	90	100	100	30	100	40
A. suum	40	40	30	30	40	20
	**C (0)**	**SH (0)**	**C (14)**	**SH (14)**	**C (28)**	**SH (28)**
**Parasite**	P %	P %	P %	P %	P %	P %
Eimeria spp.	30	40	40	10	50	20
B. coli	90	100	90	20	80	30
A. suum	80	90	100	40	100	20
T. suis	40	50	60	30	50	20
**F2**						
	**C (0)**	**CO (0)**	**C (14)**	**CO (14)**	**C (28)**	**CO (28)**
**Parasite**	P %	P %	P %	P %	P %	P %
Eimeria spp.	100	100	80	20	70	40
B. coli	70	80	80	40	90	70
A. suum	80	90	100	100	100	100
T. suis	90	90	80	80	60	50
	**C (0)**	**SH (0)**	**C (14)**	**SH (14)**	**C (28)**	**SH (28)**
**Parasite**	P %	P %	P %	P %	P %	P %
Eimeria spp.	80	80	70	10	70	30
B. coli	80	90	70	50	90	30
A. suum	90	90	80	40	100	20
T. suis	70	60	70	10	60	20

F=farm, C=control, SH=*S. hortensis*, CO=*C. officinalis.*

Table S4. The prevalence (P), at 0, 14 and 28 days in sows.

**F1**						
	**C (0)**	**CO (0)**	**C (14)**	**CO (14)**	**C (28)**	**CO (28)**
**Parasite**	P %	P %	P %	P %	P %	P %
B. coli	90	100	90	40	100	50
	**C (0)**	**SH (0)**	**C (14)**	**SH (14)**	**C (28)**	**SH (28)**
**Parasite**	P %	P %	P %	P %	P %	P %
Eimeria spp.	30	40	30	10	40	10
B. coli	90	100	70	20	80	20
A. suum	30	40	40	10	40	10
Oesophagostomum spp.	30	40	20	10	10	-
**F2**						
	**C (0)**	**CO (0)**	**C (14)**	**CO (14)**	**C (28)**	**CO (28)**
**Parasite**	P %	P %	P %	P %	P %	P %
Eimeria spp.	50	60	60	50	60	40
B. coli	60	70	90	30	80	30
A. suum	20	30	30	30	40	10
Oesophagostomum spp.	70	70	70	70	70	30
Cryptosporidium spp.	10	10	-	-	10	-
	**C (0)**	**SH (0)**	**C (14)**	**SH (14)**	**C (28)**	**SH (28)**
**Parasite**	P %	P %	P %	P %	P %	P %
Eimeria spp.	50	60	40	10	60	20
B. coli	80	80	70	20	90	30
A. suum	30	30	40	10	40	10
Oesophagostomum spp.	20	30	30	20	30	10
Cryptosporidium spp.	10	10	10	-	10	10

F=farm, C=control, SH=*S. hortensis*, CO=*C. officinalis.*
